# PRMT3 inhibitor SGC707 reduces triglyceride levels and induces pruritus in Western-type diet-fed LDL receptor knockout mice

**DOI:** 10.1038/s41598-021-04524-w

**Published:** 2022-01-10

**Authors:** Laura M. de Jong, Zhengzheng Zhang, Yvette den Hartog, Timothy J. P. Sijsenaar, Renata Martins Cardoso, Martijn L. Manson, Thomas Hankemeier, Peter W. Lindenburg, Daniela C. F. Salvatori, Miranda Van Eck, Menno Hoekstra

**Affiliations:** 1 Division of BioTherapeutics, Leiden Academic Centre for Drug Research, Leiden University, Gorlaeus Laboratories, 2333CC Leiden, The Netherlands; 2 Analytical Biosciences and Metabolomics, Division of Systems Biomedicine and Pharmacology, Leiden Academic Center for Drug Research, Leiden University, Leiden, The Netherlands; 3 Research Group Metabolomics, Leiden Center for Applied Bioscience, University of Applied Sciences Leiden, Leiden, The Netherlands; 4 Central Laboratory Animal Facility, Leiden University Medical Center, Leiden, The Netherlands; 5 Department of Clinical Sciences, Faculty of Veterinary Medicine, Utrecht University, Utrecht, The Netherlands

**Keywords:** Metabolism, Lipids

## Abstract

Protein arginine methyltransferase 3 (PRMT3) is a co-activator of liver X receptor capable of selectively modulating hepatic triglyceride synthesis. Here we investigated whether pharmacological PRMT3 inhibition can diminish the hepatic steatosis extent and lower plasma lipid levels and atherosclerosis susceptibility. Hereto, male hyperlipidemic low-density lipoprotein receptor knockout mice were fed an atherogenic Western-type diet and injected 3 times per week intraperitoneally with PRMT3 inhibitor SGC707 or solvent control. Three weeks into the study, SGC707-treated mice developed severe pruritus and scratching-associated skin lesions, leading to early study termination. SGC707-treated mice exhibited 50% lower liver triglyceride stores as well as 32% lower plasma triglyceride levels. Atherosclerotic lesions were virtually absent in all experimental mice. Plasma metabolite analysis revealed that levels of taurine-conjugated bile acids were ~ threefold increased (*P* < 0.001) in response to SGC707 treatment, which was paralleled by systemically higher bile acid receptor TGR5 signalling. In conclusion, we have shown that SGC707 treatment reduces hepatic steatosis and plasma triglyceride levels and induces pruritus in Western-type diet-fed LDL receptor knockout mice. These findings suggest that pharmacological PRMT3 inhibition can serve as therapeutic approach to treat non-alcoholic fatty liver disease and dyslipidemia/atherosclerosis, when unwanted effects on cholesterol and bile acid metabolism can be effectively tackled.

## Introduction

Non-alcoholic fatty liver disease, the accumulation of lipids in the liver also referred to as hepatic steatosis, is a pathological condition with a rapidly growing global incidence. Apart from the fact that chronic non-alcoholic fatty liver disease predisposes to the development of hepatosteatitis, which can progress to cirrhosis, liver cancer, and liver failure, hepatic steatosis is frequently paralleled by hyperlipidemia or dyslipidemia (prevalence: ~ 70%)^1^. Hyperlipidemia is an established risk factor for the development of atherosclerotic lesions, the primary underlying cause of cardiovascular disease^2^. Human subjects with non-alcoholic fatty liver disease therefore display a > 50% higher atherosclerotic cardiovascular disease susceptibility as compared to non-affected controls^3^.

Protein arginine methyltransferase 3 (PRMT3) has recently emerged from preclinical studies as a potentially interesting therapeutic target in the hepatic steatosis setting. More specifically, PRMT3 acts as co-factor for the nuclear receptor liver X receptor (LXR) in hepatocytes and thereby activates the transcription of genes involved in triglyceride synthesis^4,5^. As such, pharmacological blockade of the PRMT3 function through treatment with the selective PRMT3 inhibitor SGC707 is able to overcome the LXR agonist-induced development of hepatic steatosis in C57BL/6 wild-type mice^5^. In further support of the notion that PRMT3 inhibition can potentially serve as a novel therapeutic approach to reduce non-alcoholic fatty liver disease burden, we have shown that chronic treatment with SGC707 is also associated with markedly reduced triglyceride levels in livers of hypercholesterolemic apolipoprotein E (apoE) knockout mice after an atherogenic Western-type, high cholesterol / high fat diet challenge^6^.

Although we anticipated that the SGC707-mediated decrease in the hepatic steatosis extent would be paralleled by a reduction in atherosclerosis susceptibility in our Western-type diet-fed apoE knockout mice, SGC707-treated mice exhibited a similar atherosclerosis burden as compared to solvent control-treated mice^6^. This unexpected finding could, however, be explained by the fact that SGC707 treatment did not diminish the hyperlipidemia in Western-type diet-fed apoE knockout mice^6^. Studies by Kuipers et al. have shown that the secretion of triglyceride-rich very-low-density lipoprotein (VLDL) particles by the liver is impaired in apoE knockout mice due to the lack of hepatocyte-derived apoE^7^. As such, it can be hypothesized that the null effect of SGC707 treatment on non-fasting plasma lipid levels in apoE knockout mice is related to a relatively minor contribution of hepatic VLDL production to the hyperlipidemia in this specific mouse model. Importantly, Karasawa et al.^8^ have shown, by modulating the activity of the lipogenic transcription factor sterol regulatory element-binding protein 1c, that hepatic triglyceride synthesis does contribute significantly to plasma lipid levels and atherosclerotic lesion formation in Western-type diet-fed low density lipoprotein (LDL) receptor knockout mice, another widely-used atherosclerosis mouse model^9^. In the present study we therefore set out to assess the effect of chronic SGC707 treatment on the hepatic steatosis, hyperlipidemia and atherosclerosis extent in Western-type diet-fed LDL receptor knockout mice.

## Results

### SGC707 treatment is associated with pruritus and a decreased hepatic steatosis and hypertriglyceridemia extent in Western-type diet-fed LDL receptor knockout mice

**Figure 1:**
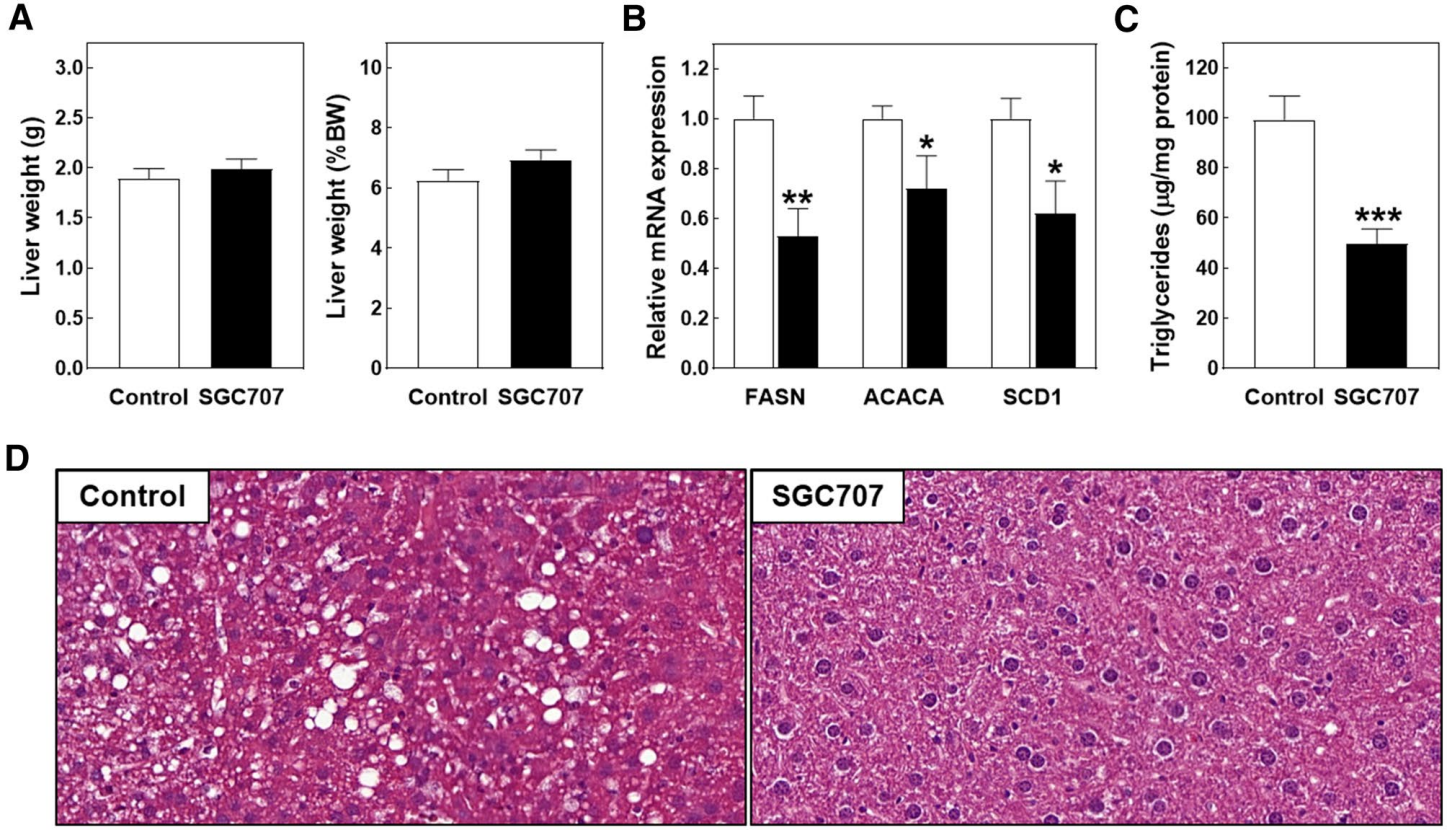
(**A**) Absolute and body weight (BW) corrected liver weights, and liver relative mRNA expression levels of lipogenic genes (**B**) and triglyceride levels (**C**) in livers of Western-type diet-fed male LDL receptor knockout mice treated with the PRMT3 inhibitor SGC707 or the solvent control. Values in the graphs represent the means + SEM of 11 mice per group. *P < 0.05, **P < 0.01, ***P < 0.001 versus control. (**D**) Representative images of hematoxylin/eosin-stained liver sections showing marked steatosis, i.e. lipid droplets, only in tissue of the control group (magnification: 400×).

The aim of this study was to verify that chronic treatment with the PRMT3 inhibitor SGC707 would inhibit hepatic triglyceride synthesis and the development of non-alcoholic fatty liver disease and, as a result, decrease the plasma hyperlipidemia extent and atherosclerosis susceptibility in Western diet-fed LDL receptor knockout mice. Unfortunately, the originally planned six-week period of high fat/high cholesterol diet feeding could not be completed as mice needed to be sacrificed halfway. The two groups of mice did not exhibit evident signs of (lethal) discomfort that warranted sacrifice, i.e. they displayed normal behaviour and activity upon handling and no apparent change in food consumption was visible between the different cages. However, 2 to 3 weeks into the study, all SGC707-treated mice developed severe pruritus, which led to the occurrence of scratching-related wounds and associated hardening of skin areas at the (lower) back. Of note, the skin at injection sites (belly area) was not visually affected nor felt different between SGC707-treated mice and control-treated mice. Since diseased / inflamed skin can impact atherosclerosis susceptibility through modifying systemic lipid metabolism and inflammation^10^, early study termination was instigated. At this point, i.e. after three weeks of combined Western-type diet feeding and compound treatment, average total body weight was not different between the two experimental groups (28.7 ± 1.0 g for SGC707-treated mice versus 30.5 ± 0.5 g for control-treated mice; *P* > 0.05). Absolute and body weight corrected liver weights were also not different between the two experimental groups (Fig. 1A). Biochemical and histological analysis on liver specimens showed that SGC707 treatment was associated with the expected decrease in lipogenic transcript levels as well as a diminished hepatic steatosis extent. More specifically, hepatic relative mRNA expression levels of fatty acid synthase (FASN), acetyl-CoA carboxylase 1 (ACACA), and stearoyl-CoA desaturase 1 (SCD1) were respectively 47% (*P* < 0.01), 28% (*P* < 0.05), and 38% (*P* < 0.05) lower after SGC707 treatment (Fig. 1B). Livers from SGC707-treated mice contained 50% less triglycerides as compared to those from control-treated mice (*P* < 0.001; Fig. 1C). The degree of steatosis observed in hematoxylin/eosin-stained liver sections from control-treated mice was already modest because of the short duration of study. However, in line with the reduction in hepatic triglyceride accumulation / steatosis upon pharmacological PRMT3 inhibition, lipid droplets were virtually absent in liver sections from SGC707-treated mice (Fig. 1D).

**Figure 2:**
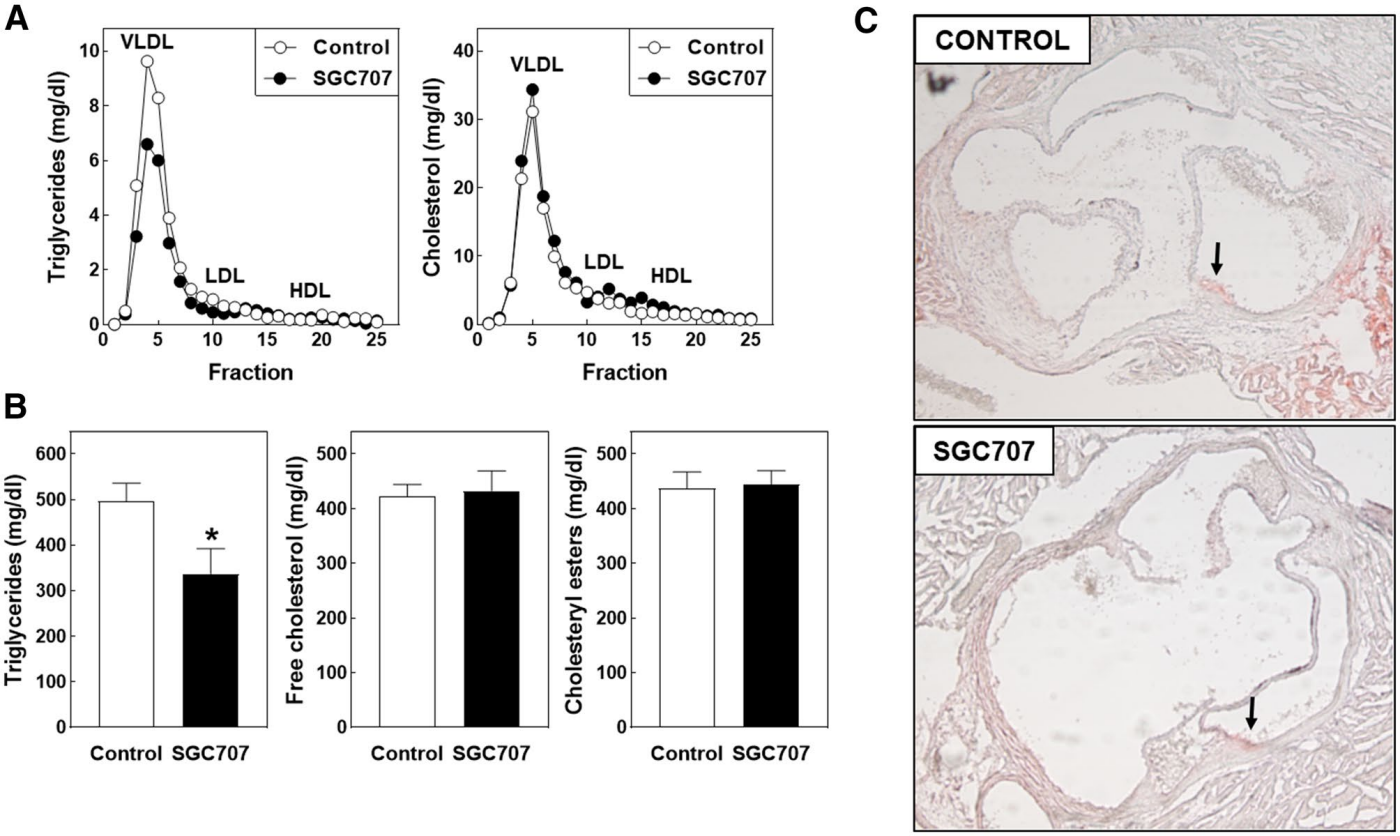
(**A**) The distribution of cholesterol and triglycerides over very-low-density lipoprotein (VLDL), low-density lipoprotein (LDL), and high-density lipoprotein (HDL) fractions and (**B**) plasma total lipid levels in Western-type diet-fed male LDL receptor knockout mice treated with the PRMT3 inhibitor SGC707 or the solvent control. Values in the graphs represent the means + SEM of 11 mice per group. *P < 0.05 versus control. (**C**) Representative images of Oil red O-stained aortic root sections with arrows indicating the minimal vascular lipid deposition (magnification: 50×).

In line with the notion that hepatic synthesis and packaging of fatty acids in triglycerides within VLDL particles is reduced upon PRMT3 inhibition, a reduction in the plasma VLDL-triglyceride concentration (Fig. 2A) was observed in SGC707-treated mice that translated into a 32% reduction (*P* < 0.05) in plasma total triglyceride levels (Fig. 2B). Plasma concentrations of free cholesterol and cholesteryl esters or the distribution of cholesterol over the different lipoprotein fractions were, however, not affected by SGC707 treatment (Fig. 2A, B). As can be appreciated from the representative images in Fig. 2C, aortic root areas of mice from the two experimental groups contained a minimal amount of plaque as a result of the limited time of Western-type diet feeding. Therefore, the overall effect on atherosclerosis outcome of the SGC707 treatment-induced change in the plasma lipid profile could not really be determined.

### SGC707 treatment is associated with a mild reduction in white adipocyte size in Western diet-fed LDL receptor knockout mice

**Figure 3:**
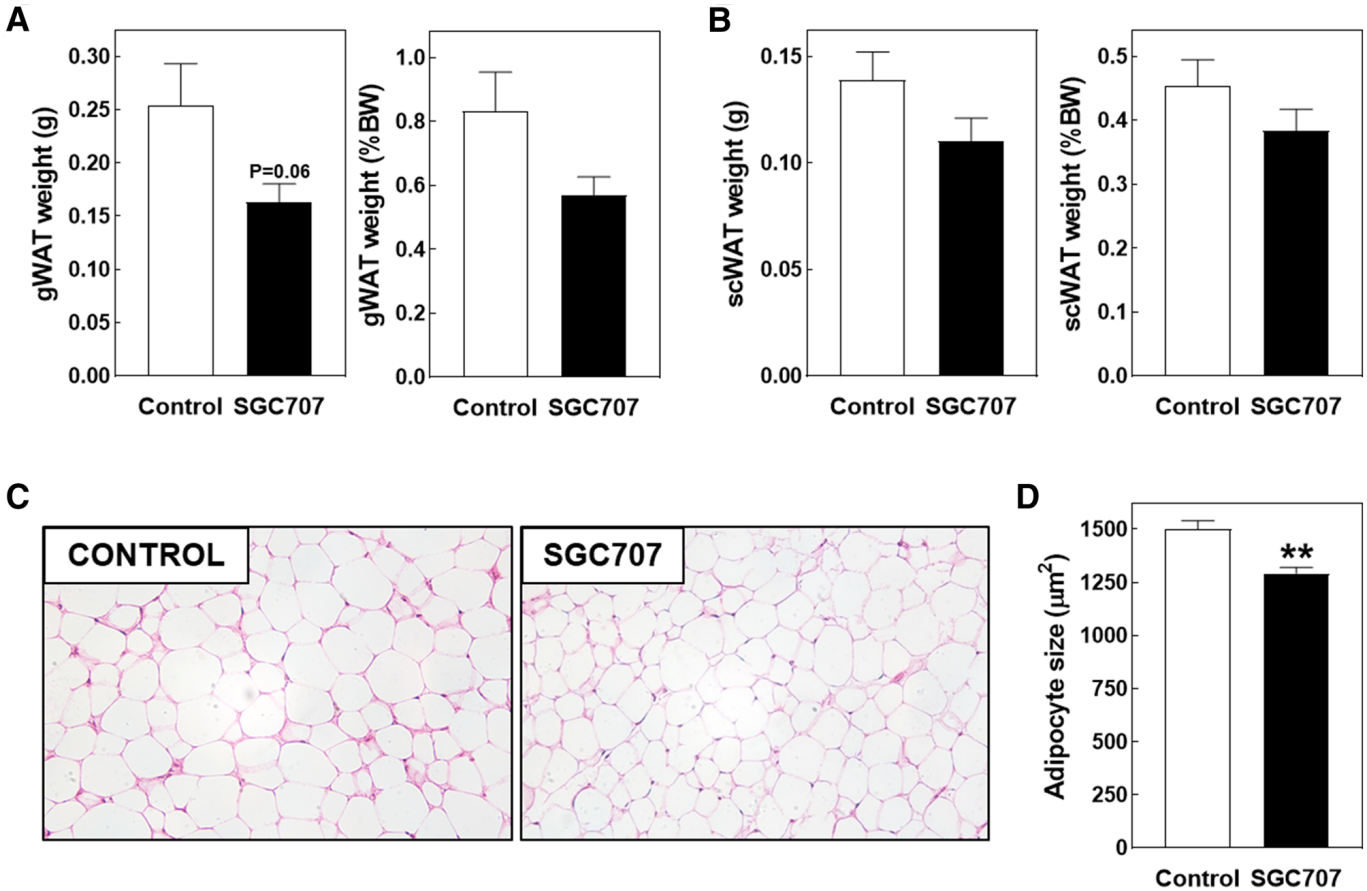
Absolute and body weight (BW) corrected gonadal (**A**) and subcutaneous (**B**) white adipose tissue weights and white adipocyte sizes in gonadal adipose tissue pads (**D**) in Western-type diet-fed male LDL receptor knockout mice treated with the PRMT3 inhibitor SGC707 or the solvent control. Values in the graphs represent the means + SEM of 11 mice per group. **P < 0.01 versus control. (**C**) Representative images of hematoxylin/eosin-stained gonadal white adipose tissue sections (magnification: 200×).

Our previous studies in hypercholesterolemic apoE knockout mice showed that SGC707 treatment was not only associated with a reduction in the hepatic steatosis extent but also with a decrease in gonadal white adipose tissue adipocyte size^6^. Non-significant reductions in absolute and body weight corrected weights of both gonadal and subcutaneous white adipose tissue depots were observed in response to SGC707 treatment in our current LDL receptor knockout experimental setting (Fig. 3A, B). Histological analysis of haematoxylin/eosin-stained sections revealed that adipocytes present in the gonadal white adipose tissue were slightly smaller after SGC707 treatment (Fig. 3C). Average white adipocyte areas were respectively 1293 ± 36 µm^2^ in SGC707-treated mice versus 1503 ± 47 µm^2^ in control-treated mice (*P* < 0.01; Fig. 3D). No browning of gonadal white adipose tissue was seen in any of the tissue sections (Fig. 3C). Gene expression levels of PRMT3 in subcutaneous adipose tissue specimens of control-treated mice were at the border of reliable detection (average Ct: 34.3 ± 1.4). A direct metabolic action of SGC707 in white adipocytes is therefore considered unlikely.

### SGC707 treatment indirectly stimulates scratching leading to skin damage

**Figure 4:**
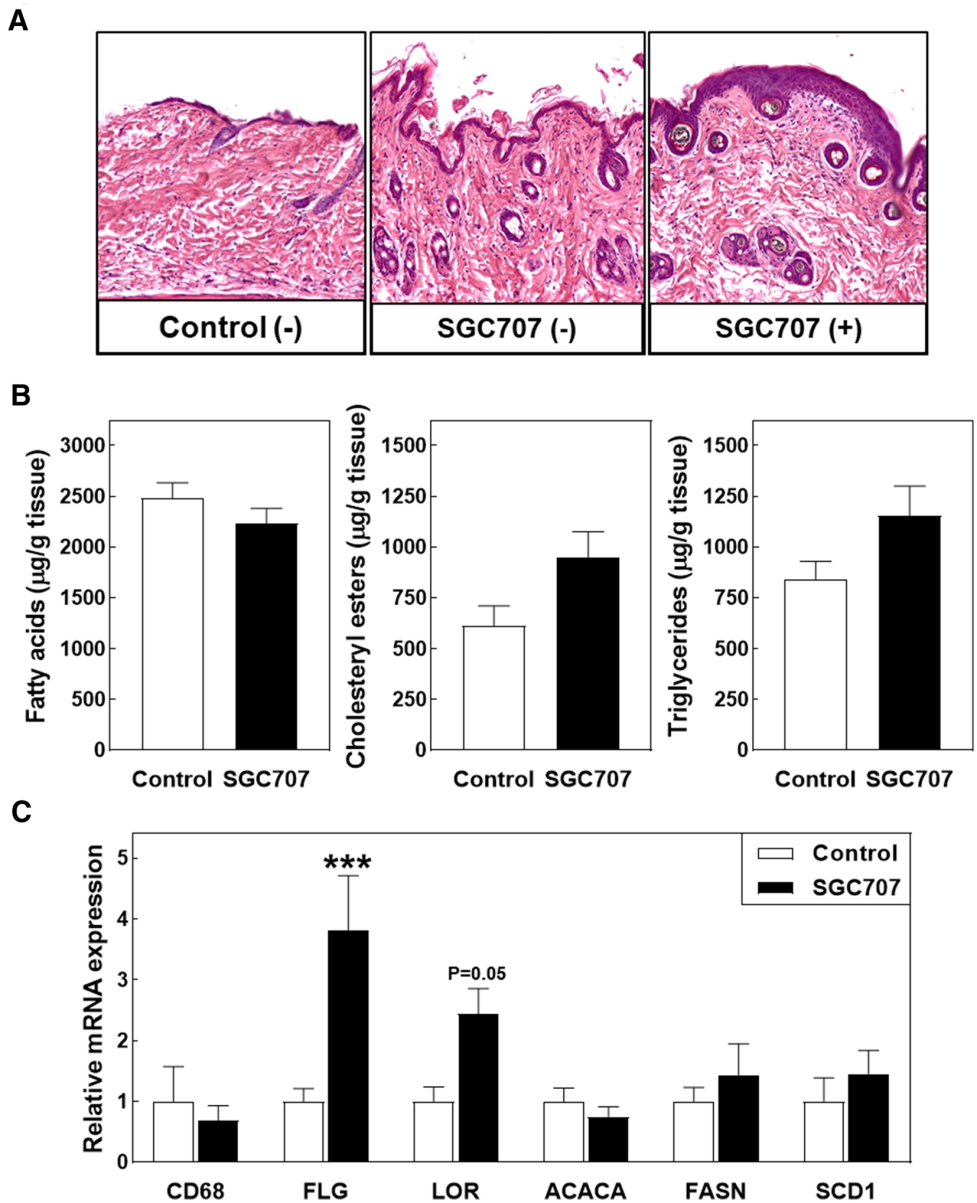
(**A**) Representative images of hematoxylin/eosin-stained sections of skin with (+) or without (−) scratching wounds (magnification: 40×), and lipid (**B**) and gene expression (**C**) levels in skin of Western-type diet-fed male LDL receptor knockout mice treated with the PRMT3 inhibitor SGC707 or the solvent control. Values in the graph represent the means + SEM of 6 mice per group. ***P < 0.01 versus control.

Skin of the different experimental mice was collected at sacrifice to uncover the reason behind the unexpected pruritus development. As can be seen from the representative images of haematoxylin/eosin-stained paraffin sections in Fig. 4A, visually non-affected skin parts from SGC707-treated and control-treated mice exhibited a normal dermis morphology and contained an equal number of epidermal layers covered by a thin stratum corneum. Epidermal hyperplasia was, however, detected in scratched skin parts obtained from SGC707-treated mice (Fig. 4A). Previous studies in apoE knockout mice and scavenger receptor BI knockout mice have highlighted that changes in tissue lipid levels as a result of the genetic hyperlipidemia can underlie the development of pathological skin phenotypes, i.e. disrupt epidermal layer organization and impair skin barrier function and/or induce the accumulation of inflammatory cells such as macrophages and T cells^11–13^. However, tissue lipid extraction and quantification revealed that SGC707 treatment did not significantly impact skin fatty acid, cholesteryl ester, and triglyceride stores (Fig. 4B). The clusters of immune cells previously detected by Arnaboldi et al. in the dermis of hyperlipidemic apolipoprotein A1 / E double knockout mice^13^ were also not observed in skin sections from our SGC707-treated and control-treated mice (Fig. 4A). Gene expression analysis validated that T cells, B cells, and neutrophils were present at very low to non-detectable levels in whole back skin specimens from both SGC707-treated and control-treated mice (CD3, CD4, CD8, CD19, and Ly6G: average Ct > 35). Macrophage marker CD68 transcripts could be reliably detected—albeit at low levels—in the mouse skin samples (average Ct: 34–35), but levels were not different between the two groups of mice (Fig. 4C). It thus appears that the pathological skin phenotype seen upon SGC707 treatment did not arise from major local changes at the lipid or inflammatory level. Notably, significant upregulation of the keratinocyte terminal differentiation marker filaggrin (FLG; + 308%; *P* < 0.001) and a clear trend towards an increase (+ 132%; *P* = 0.05) in transcript levels of the epidermal differentiation complex protein loricrin (LOR) was observed in skin from SGC707-treated mice (Fig. 4C). An active keratinocyte stress response was thus apparently ongoing in the skin of SGC707-treated animals, probably to protect the skin against scratching-associated disruption of the functional barrier. Gene expression analysis did not reveal a significant difference in relative mRNA expression levels of the lipogenic PRMT3 target genes FASN, ACACA, or SCD1 between the two treatment groups (Fig. 4C). Since PRMT3 gene expression levels were too low to be reliably detected in all skin samples (Ct > 35), it is anticipated that the pruritus induction was not due to an effect of SGC707 locally within the skin.

### SGC707 treatment is associated with increased plasma bile acid levels and TGR5 activation

**Figure 5:**
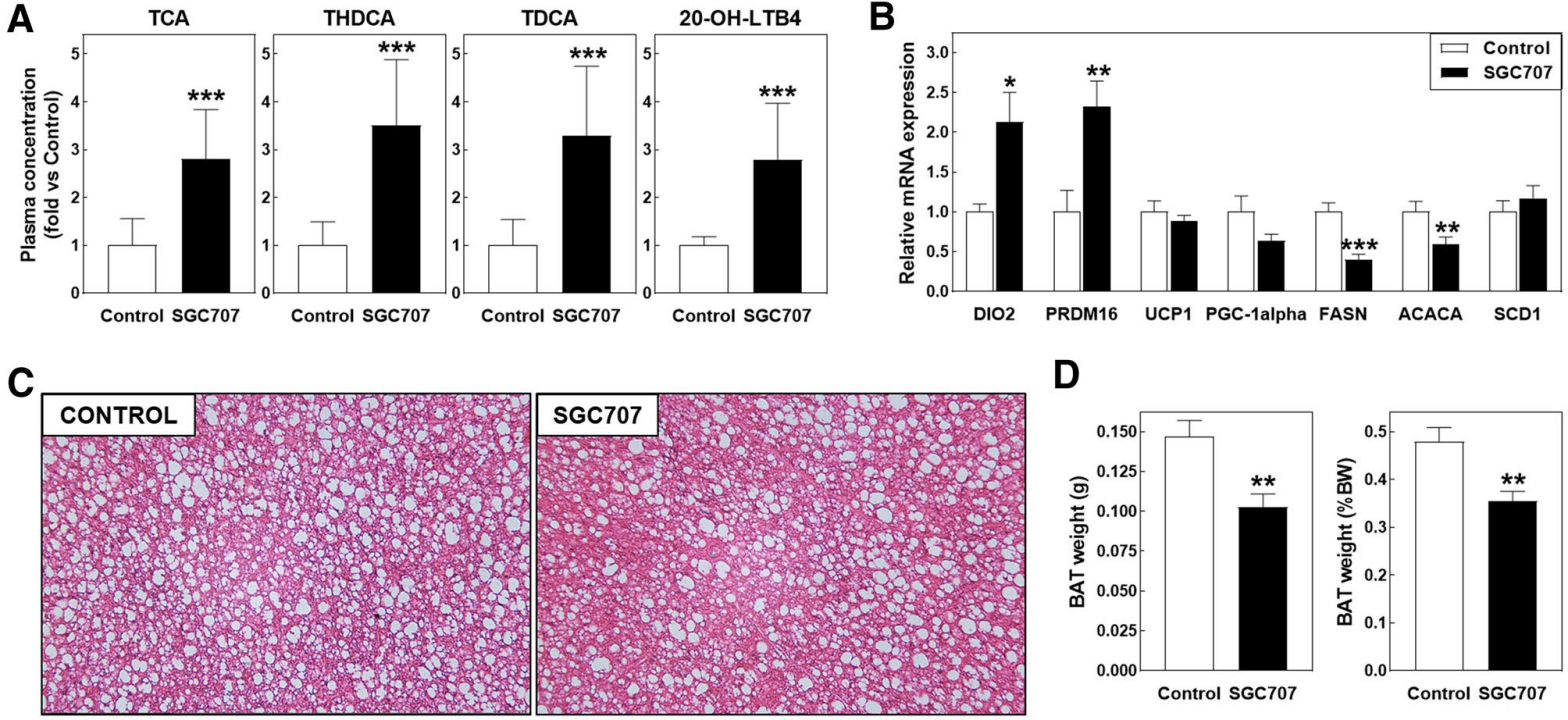
(**A**) Plasma concentrations of the bile acid species TCA, THDCA, and TDCA and 20-hydroxy-leukotriene B4 (20-OH-LTB4) and brown adipose tissue gene expression levels (**B**) and weights (**D**) in Western-type diet-fed male LDL receptor knockout mice treated with the PRMT3 inhibitor SGC707 or the solvent control. Values in the graphs represent the means + SEM of 5 plasma pools (panel A) or 11 mice per group (panels B and D). *P < 0.05, **P < 0.01, ***P < 0.001 versus control. (**D**) Representative images of hematoxylin/eosin-stained brown adipose tissue sections (magnification: 200×).

Ultra-high-performance liquid chromatography-tandem mass spectrometry was employed to uncover potential changes in the plasma metabolome that could contribute to the SGC707 treatment-associated change in white adipocyte and skin phenotype. A full overview of the plasma quantification results from the total of 193 bio-active metabolites can be found in Supplemental Table 1. Two-way ANOVA suggested that SGC707 treatment did not execute a significant effect on the overall plasma metabolite profile (metabolite effect: *P* < 0.001; SGC707 treatment effect: *P* = 0.15; interaction effect: *P* < 0.001). However, the two-way ANOVA Bonferroni post-test revealed that relative plasma levels of 4 metabolites were significantly different between SGC707-treated and control-treated mice (Fig. 5A). More specifically, SGC707 treatment was associated with marked increases in plasma levels of the bile acids taurocholic acid (TCA; + 180%; *P* < 0.001), taurohyodeoxycholic acid (THDCA; + 251%; *P* < 0.001), and taurodeoxycholic acid (TDCA; + 229%; *P* < 0.001). In addition, a 179% rise (*P* < 0.001) in relative plasma levels of 20-hydroxy-leukotriene B4 (20-OH-LTB4) was detected in response to SGC707 treatment.

Previous studies have shown that relatively high levels of bile acids in plasma can activate the Takeda G protein-coupled receptor 5/G-protein-coupled bile acid receptor (TGR5 / GPBAR1) that induces itching and scratching behaviour^14,15^ and promotes energy expenditure by modulating brown adipose tissue activity^16^. We therefore determined whether TGR5 activity in skin and brown adipose tissue was enhanced upon SGC707 treatment by measuring relative expression levels of the TGR5 target gene thyroid hormone deiodinase 2 / type II iodothyronine deiodinase (DIO2) that converts inactive thyroid hormone T4 into its active counterpart T3. DIO2 expression levels could be reliably detected (Ct < 35) in 4 out of 6 skin samples from SGC707-treated mice and only in 1 out of 6 skin samples from control-treated mice (Chi-square test P value: 0.079). As such, no clear conclusion can be drawn regarding the effect of SGC707 treatment on skin TGR5 activity. Importantly, a marked 113% increase (*P* < 0.05) in relative DIO2 mRNA expression levels was detected in brown adipose tissue (Fig. 5B). In vitro studies by Yau et al. have suggested that an increase in brown adipocyte T3 concentrations, as a result of the observed increase in DIO2 levels, can theoretically lead to a concomitant rise in gene expression levels of uncoupling protein 1 (UCP1), PR/SET Domain 16 (PRDM16), and peroxisome proliferator-activated receptor-gamma coactivator-1alpha (PGC-1alpha)^17^. Brown adipose tissue PRDM16 levels did significantly increase upon SGC707 treatment (+ 132%; *P* < 0.01), whilst UCP1 and PGC-1alpha levels remained unaltered (Fig. 5B). PRMT3 expression gene expression levels were readily detectable in brown adipose tissue from control-treated mice (average Ct: 30.0 ± 0.2). In accordance with a potential role for PRMT3 in the transcriptional control of lipogenesis also locally in brown adipocytes, relative mRNA expression levels of FASN (−60%; *P* < 0.001) and ACACA (−41%; *P* < 0.01), but not SCD1, were decreased in SGC707-treated mice (Fig. 5B). The changes in the gene expression profile were not paralleled by a major difference in the general brown adipose tissue morphology as judged from haematoxylin/eosin-stained paraffin sections. The size of the brown adipocyte lipid droplets did not seem to markedly differ between adipose tissue from SGC707-treated and control-treated mice (Fig. 5C). The total weight of the brown adipose tissue depots was—however—significantly decreased upon SGC707 treatment, which remained significant after correcting the tissue weight for total body weight (−30% and −26%, respectively; *P* < 0.01 for both; Fig. 5D).

### SGC707 treatment is associated with hepatic free cholesterol accumulation and a concomitant increase in bile acid synthesis

**Figure 6:**
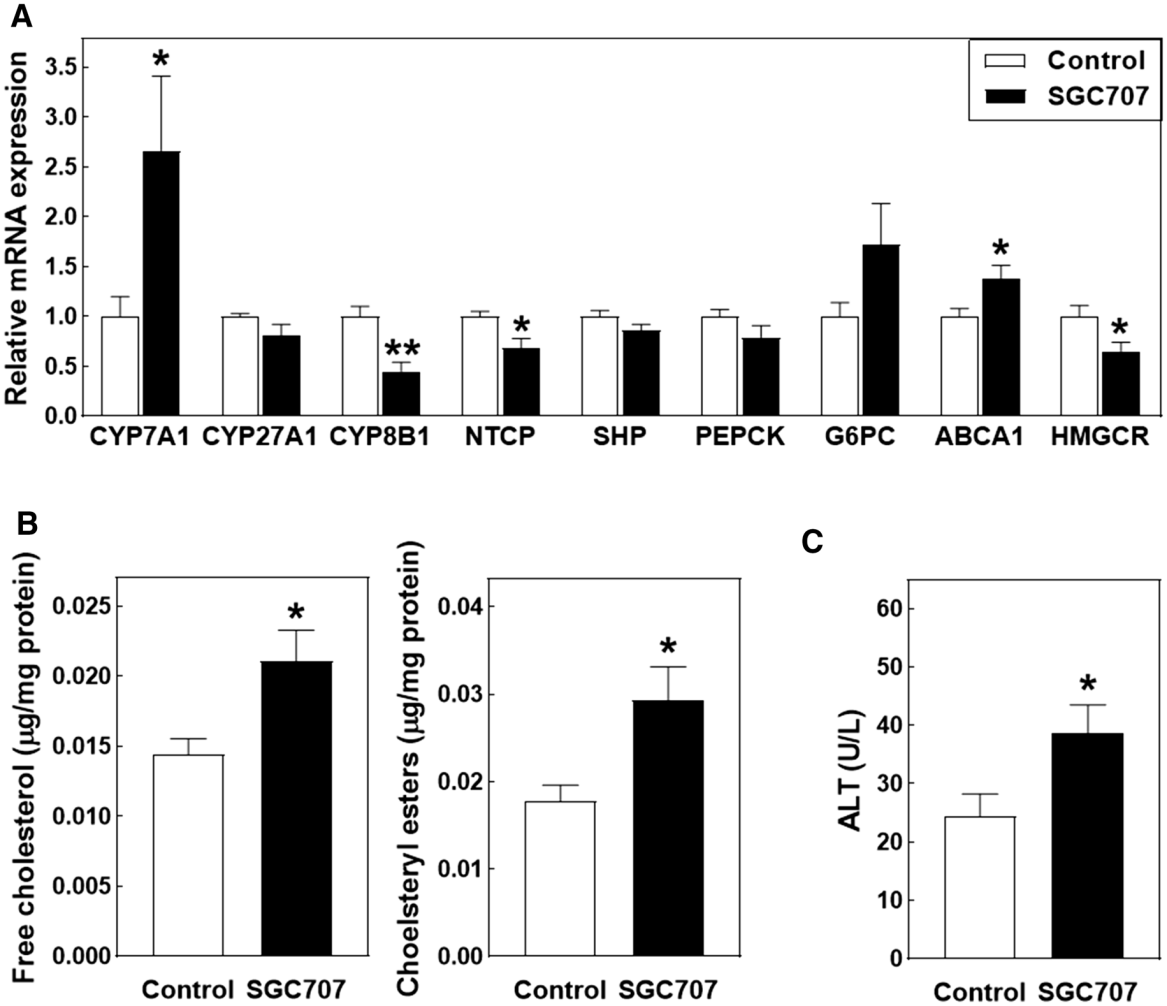
Hepatic gene expression (**A**) and free cholesterol and cholesteryl ester (**B**) levels and plasma ALT levels (**C**) in Western-type diet-fed male LDL receptor knockout mice treated with the PRMT3 inhibitor SGC707 or the solvent control. Values in the graphs represent the means + SEM of 9–11 mice per group. *P < 0.05 versus control.

The liver is an important regulator of the plasma bile acid pool as it (1) is able to newly synthesize bile acids from cholesterol and (2) facilitates the clearance of bile acids from the blood circulation. Gene expression analysis revealed that SGC707 treatment was associated with a 166% increase (*P* < 0.05; Fig. 6A) in hepatic mRNA levels of cholesterol 7alpha-hydroxylase (CYP7A1), the rate-limiting enzyme in classical bile acid synthesis in mice. No change was detected in hepatic gene expression levels of sterol 27-hydroxylase (CYP27A1) that directs bile acid synthesis to the generation of chenodeoxycholic acid (Fig. 6A). In contrast, mRNA expression levels of the cholic acid synthesis driving enzyme sterol 12-alpha-hydroxylase (CYP8B1) were significantly reduced in livers of SGC707-treated mice as compared to control-treated mice (−55%; *P* < 0.01; Fig. 6A). Transcript levels of the bile acid uptake protein sodium/bile acid cotransporter (SLC10A1 / NTCP) were also decreased in SGC707-treated mice (−32%; *P* < 0.05; Fig. 6A). It can therefore be suggested that the SGC707 treatment-associated accumulation of bile acids in the plasma compartment was primarily due to the markedly increased de novo bile acid synthesis, but perhaps also resulting from diminished hepatic bile acid clearance.

Hepatic bile acid synthesis by CYP7A1 is normally subject to negative feedback, which is mediated by a concerted action of the nuclear receptors farnesoid X receptor (FXR/BAR; NR1H4) and small heterodimer partner (SHP; NR0B2)^18,19^. No significant differences were seen in levels of small heterodimer partner (SHP) or its downstream target genes phosphoenolpyruvate carboxykinase (PEPCK) and glucose 6-phosphatase alpha (G6PC) (Fig. 6A), arguing against a major impact of SGC707 treatment on the activity of the FXR → SHP pathway.

In mice, CYP7A1 transcription is stimulated upon oxysterol-mediated activation of LXR to facilitate removal of excess free cholesterol from hepatocytes^20^. In accordance with a generally higher LXR activation in livers from SGC707-treated mice, a 38% increase (*P* < 0.05; Fig. 6A) was also detected in relative mRNA expression levels of the LXR target gene ATP-binding cassette transporter A1 (ABCA1) that mediates the efflux of cholesterol from apolipoprotein A1 to accommodate HDL particle formation. Tissue lipid analysis validated that livers from SGC707-treated mice contained relatively high levels of both free cholesterol (+ 47%; *P* < 0.05) and cholesteryl esters (+ 65%; *P* < 0.05) (Fig. 6B). Cellular free cholesterol overload is an established inducer of endoplasmic reticulum stress, mitochondrial dysfunction and hepatocyte toxicity^21^. In accordance, plasma levels of the hepatotoxicity marker ALT were somewhat elevated in response to SGC707 treatment (+ 47%; *P* < 0.05; Fig. 6C), but remained within the normal physiological range for mice (25–60 U/L)^22^. In further support of an enriched regulatory free cholesterol pool in hepatocytes of SGC707-treated mice, compensatory downregulation was detected in mRNA expression levels of the key cholesterol synthesis gene 3-hydroxy-3-methyl-glutaryl-coenzyme A reductase (HMGCR) (−35%; *P* < 0.05; Fig. 6A).

## Discussion

**Figure 7:**
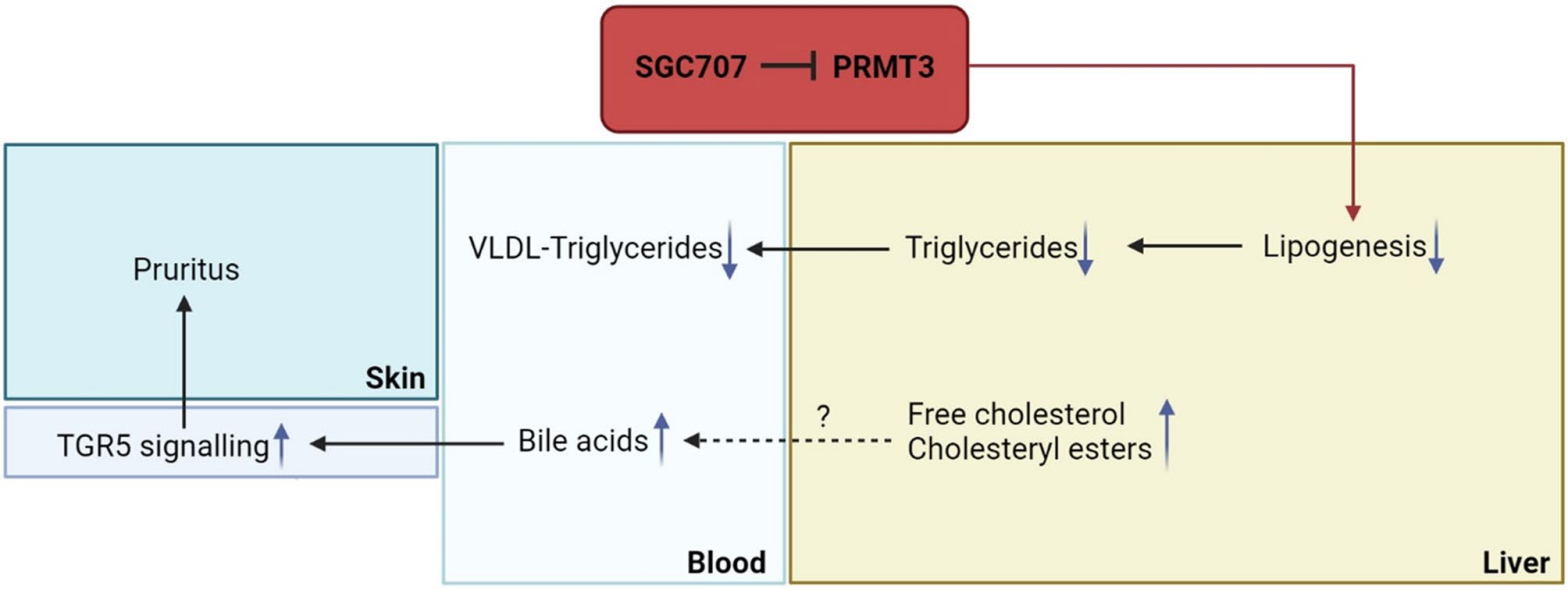
Schematic overview of the effect of PRMT3 inhibitor SGC707 treatment in LDL receptor knockout mice. Inhibition of the PRMT3 activity by SGC707 is associated with a decrease in transcript levels of gene involved in lipogenesis and a parallel reduction in hepatic triglyceride stores and plasma VLDL-triglyceride levels. Probably as result of decreased cholesterol elimination via VLDL particle secretion, livers of SGC707-treated mice accumulate free cholesterol and cholesteryl esters and generate more bile acids. The associated increase in the plasma bile acid pool coincides with enhanced systemic TGR5 activation, which can underlie the unexpected pruritus development in SGC707-treated mice.

As can be appreciated from the schematic results overview in Fig. 7, in the present study we have shown that PRMT3 inhibition does not only reduce hepatic triglyceride accumulation but also decreases VLDL-triglyceride levels in Western-type diet-fed LDL receptor knockout mice. In addition, we found that SGC707 treatment was associated with hepatic cholesterol and plasma bile acid accumulation and the development of severe pruritus. As a result, the overall effect of SGC707 treatment on atherosclerosis susceptibility in Western-type diet-fed LDL receptor knockout mice remains unknown.

The finding that SGC707 treatment reduced the gene expression of lipogenesis mediators FASN, ACACA, and SCD1 as well as liver triglyceride levels is in accordance with our working hypothesis, originally derived from studies by Kim et al.^4^, that hepatocyte PRMT3 acts as a selective lipogenic transcriptional co-activator of the nuclear receptor LXRalpha^5^. Previous studies by Terasaka et al. have shown that 8 week treatment of atherogenic diet-fed LDL receptor knockout mice with the synthetic LXR agonist T0901317 does not influence plasma total cholesterol levels, whilst it does increase the overall plasma triglyceride exposure^23^. That SGC707 treatment in our current study specifically lowered the triglyceride content of VLDL particles without impacting plasma total cholesterol levels supports the notion that PRMT3 inhibition effectively disrupts hepatic LXR functionality. In addition, it concurs with the earlier observation by Grefhorst et al. that hepatic LXR activation is associated with hepatic steatosis development and the formation of relatively large, triglyceride-rich VLDL particles^24^.

A striking observation was that SGC707 induced pruritus and development of scratching-associated skin wounds in our current LDL receptor knockout setting, a phenotype that was previously not seen when we subjected atherogenic diet-fed apoE knockout mice to the same SGC707 treatment regimen^6^. Since activation of the bile acid receptor TGR5 has been linked to itch induction in mice^15,25^ and a higher relative mRNA expression level of the TGR5 target gene DIO2 was detected in brown adipose tissue of our SGC707-treated mice, we consider plasma bile acid accumulation the driving force behind the unexpected skin scratching. However, it cannot be excluded that other mechanisms also contributed to the pathological itch induction. Of note, the bile acid-mediated TGR5 activation can also explain the marked decrease in hepatic CYP8B1 transcript levels in the context of higher CYP7A1 expression levels and an unchanged FXR activation status^26^. The increased bile acid signalling in brown adipose tissue was not paralleled by UCP1 expression upregulation or the presence of smaller adipocytes, as could be anticipated from previous studies by Zietak et al. in cholic acid-treated mice^27^. However, this may be related to the relatively short total duration of the current study, i.e. 3 weeks versus 9 weeks in the studies by Zietak et al.^27^. We did observe that the brown adipose tissue mass was reduced upon SGC707 treatment. Additional studies in cultured brown adipocytes are required to dissociate whether this latter effect is due to the rise in plasma bile acid levels, similarly as seen in NTCP knockout mice^28^, or can rather be attributed to local PRMT3 activity inhibition by SGC707. The increase in plasma bile acid concentrations appeared to be secondary to the LXR-driven stimulation of CYP7A1-mediated hepatocyte bile acid synthesis in response to cellular free cholesterol accumulation. Importantly, SGC707 treatment did not impact hepatic cholesterol levels in atherogenic diet-fed apoE knockout mice^6^. As such, we anticipate that the increase in hepatic (free) cholesterol levels ultimately underlies the plasma bile acid accumulation and associated pruritus development. That hepatic cholesterol levels were increased in response to SGC707 treatment in our LDL receptor knockout mice (but not in apoE knockout mice) can possibly be explained by the fact that the basal elimination of cholesterol from hepatocytes via the VLDL pathway is particularly high in these mice^7^. SGC707 treatment diminishes fatty acid and triglyceride formation and the generation of triglyceride- and cholesterol-containing VLDL particles, which is therefore expected to directly result in reduced efflux of cholesterol from the liver and culminate in the observed hepatic free cholesterol and cholesteryl ester accumulation. A clear limitation of our studies is that the contribution of PRMT3 to hepatic lipogenesis and VLDL production it solely based upon effects of SGC707 treatment measured at the transcriptional level. Dedicated studies, i.e. using stable isotopes, are therefore warranted to show the quantitative effect of SGC707-mediated PRMT3 inhibition on (hepatic) fatty acid and cholesterol fluxes.

In conclusion, we have shown that SGC707 treatment reduces the hepatic steatosis extent and lowers VLDL-triglyceride levels and induces pruritus in Western-type diet-fed LDL receptor knockout mice. Relatively high concentrations of plasma triglycerides are associated with an increased risk of developing heart failure and ischemic stroke as well as non-Alzheimer dementia in the human general population^29,30^. Pharmacological inhibition of PRMT3 activity can thus theoretically serve as a therapeutic approach to battle both the development of hepatic steatosis and age-related cardiovascular and cognitive pathologies. However, more insight into the in vivo pharmacology of SGC707 and tissue-specific physiological roles of PRMT3 is needed to potentially be able to overcome the unwanted hepatic cholesterol and plasma bile acid accumulation and development of pruritus upon PRMT3 inhibition.

## Materials and methods

### Mice

Twelve week old male LDL receptor knockout mice on a C57BL/6 background, originally obtained from the Jackson Laboratory and bred in house, were group-housed with 2 to 5 mice per cage in filter-top cages under standard climate conditions (22 °C; 12H light / 12H dark) within the animal facility of the Gorlaeus Laboratories. To induce the development of atherosclerotic lesions, mice were fed a Western-type diet containing 0.25% cholesterol and 15% cocoa butter (SDS, Sussex, UK) ad libitum. Mice were randomly distributed over two treatment groups to receive either 3 intraperitoneal injections with SGC707 (0.3 mg per injection representing a final dose of ~ 10 mg/kg body weight; N = 15) or a similar amount of solvent control (10 µl DMSO; N = 15) per week during the Western-type diet feeding period. During the study initiation, four control mice and four SGC707-treated mice developed severe fighting wounds, resulting in elimination of these mice from further analysis. Three weeks into the study, all 11 mice in the SGC707 treatment group displayed unexpected, continuous skin scratching. Given the associated development of skin wounds, a consultant from the Animal Welfare Body advised to terminate the study. For this purpose, the remaining 22 mice (11 SGC707-treated mice and 11 solvent control-injected mice) were euthanized using CO_2_ in the ad libitum fed (non-fasted) state. Blood was drawn by cardiac puncture and stored in EDTA-coated vials for plasma collection. Subsequently, shaved back skin, heart, liver, kidney, intestine, and gonadal and subcutaneous white adipose tissue and brown adipose tissue specimens were collected from each mouse and stored at −20 °C or in a formalin solution until further analysis. Animal experiments were performed in accordance with the ARRIVE guidelines and approved by the Dutch Central Commission for Animal experimentation (Centrale Commissie voor Dierproeven) according to the Dutch Law on laboratory animal experimentation and the EU Directive 2010/63/EU.

### Tissue lipid extraction and quantification

Triglycerides were extracted from 50 mg of homogenized liver tissue or 75–150 mg skin tissue in 500 μl of Nonidet™P40 Substitute. The homogenate was subjected to 3 cycles of heating until 90 °C and cooling on ice to solubilize the triglycerides. Subsequently, all insoluble material was removed by centrifugation. An enzymatic colorimetric assay (Roche Diagnostics) was used to determine triglycerides concentrations. Cholesterol and fatty acids were extracted from tissues using the Folch extraction method^31^. To determine the concentration of free cholesterol and cholesteryl esters in liver and skin, an enzymatic colorimetric assay was performed as described by Out et al.^32^. An enzymatic colorimetric assay (Roche Diagnostics) was used to determine fatty acids concentrations in the skin.

Tissue lipid contents were corrected for the total protein content (liver) or corrected for the tissue weight (skin). Protein concentrations were determined using a Pierce™ BCA Protein Assay Kit (ThermoFisher Diagnostics, Waltham, MA, USA). Precipath (Roche Diagnostics) was used as a reference standard in the colorimetric assays.

### Plasma analyses

The concentrations of total cholesterol and triglycerides were determined in whole plasma samples of each mouse with aid of the enzymatic colorimetric assays as described above. The distribution of cholesterol and triglycerides over the different lipoproteins was analysed by fractionation of plasma pools containing equal volumes of all mice in each treatment group using a Superose 6 column (3.2 × 300 mm, Smart-system, Pharmacia). In addition, plasma pools containing samples from 2 mice from per treatment group (total n = 4/5 of pooled samples) were run in an untargeted LC–MS/MS lipidomics analysis as set up by Schoeman et al.^33^. The metabolite panel consisted of > 100 different species, including essential fatty acids, bile acids, isoprostanes, sphingoid mediators, and lysophosphatidic acids. Integrated peak sizes of each individual metabolite were expressed relative to those of the control group. Plasma alanine aminotransferase (ALT) levels were measured with the ALT activity kit from Sigma according to the protocol of the supplier.

### Histological analysis

Formalin-fixed liver, skin, white and brown adipose tissue was embedded in paraffin and sectioned using a RM2235 rotary microtome (Leica Ltd., Cambridge, UK). Liver and white/brown adipose tissue specimens were cut at 5 μm thickness, whereas skin samples were cut at 8 μm thickness. Sections were stained with hematoxylin and eosin to evaluate the hepatic steatosis extent and adipose tissue and skin structure. Representative images were obtained using a Leica image analysis system. To quantify the average adipocyte area in gonadal white adipose tissue specimens, fifteen representative photos of tissue sections from individual mice were taken with a 20 × objective, enabling size quantification of at least 200 adipocytes per mouse using CellProfiler 3.1.8 (Cambridge, UK).

### Analysis of gene expression by real-time quantitative PCR

Total RNA was isolated from the different tissues as previously described by Chomczynski and Sacchi^34^. The RNA quality and concentration was measured using a Nanodrop Spectrophotometer. cDNA was synthesized from 1 μg of total RNA with the aid of M-MuLV reverse transcriptase. Quantitative gene expression analysis was performed on an Applied Biosystems 7500 apparatus using SYBR Green technology, as described^35^. Peptidylpropyl isomerase A (PPIA) and beta-actin (ACTB) were used as standard housekeeping genes for the liver and adipose tissue analyses and PPIA, tyrosine 3-monooxygenase / tryptophan 5-monooxygenase activation protein zeta (YWHAZ), eukaryotic translation elongation factor 2 (EEF2), and glyceraldehyde-3-phosphate dehydrogenase (GAPDH) for analysis of the skin specimens, respectively. Genes were identified as being reliably expressed when they displayed a cycle threshold (Ct) value of < 35 in all samples. For calculation purposes, a Ct value of 40 was applied when genes did not show any expression.

### Statistical analysis

Statistical analyses were performed using GraphPad Prism 5.0 (GraphPad Software Inc., San Diego, CA, USA). A two-tailed Student’s t-test or Chi-square without Yates correction was used to calculate the significance of differences between two groups. Plasma metabolite profiles were tested for significant differences using a two-way analysis of variance (ANOVA) with Bonferroni post-test. In all statistical comparisons, a *P*-value < 0.05 was considered statistically significant.

## Supplementary Information

Supplementary Information.
