# Supplementary material for: PRMT3 inhibitor SGC707 reduces triglyceride levels and induces pruritus in Western-type diet-fed LDL receptor knockout mice
Source: Sci Rep. 2022 Jan 10;12:483. doi: 10.1038/s41598-021-04524-w (PMC8748717; doi:10.1038/s41598-021-04524-w)
Supplement: Supplementary file 1 — Supplementary Information. [file 41598_2021_4524_MOESM1_ESM.pdf]

Supplemental Table 1: Plasma metabolite profile of Western-type diet-fed male LDL receptor knockout mice treated with the PRMT3 inhibitor SGC707 or the solvent control.

METABOLITE SPECIES	CONTROL	SGC707	P-value
COR	1,00 ± 0,19	0,97 ± 0,11	N.S.
a-LEA	1,00 ± 0,23	0,84 ± 0,11	N.S.
POEA	1,00 ± 0,22	0,61 ± 0,08	N.S.
PDEA	1,00 ± 0,27	0,81 ± 0,12	N.S.
LEA	1,00 ± 0,08	1,06 ± 0,04	N.S.
AEA	1,00 ± 0,16	0,68 ± 0,04	N.S.
O-AEA	1,00 ± 0,16	0,62 ± 0,05	N.S.
DHEA	1,00 ± 0,18	1,31 ± 0,29	N.S.
2-AG	1,00 ± 0,14	1,23 ± 0,15	N.S.
1-AG	1,00 ± 0,18	1,16 ± 0,17	N.S.
2-LG	1,00 ± 0,15	1,29 ± 0,15	N.S.
1-LG	1,00 ± 0,13	0,92 ± 0,16	N.S.
PEA	1,00 ± 0,06	0,99 ± 0,12	N.S.
DGLEA	1,00 ± 0,11	1,07 ± 0,15	N.S.
2-AGE	1,00 ± 0,33	1,16 ± 0,55	N.S.
2-OG	1,00 ± 0,13	0,99 ± 0,18	N.S.
1-OG	1,00 ± 0,07	0,95 ± 0,26	N.S.
SEA	1,00 ± 0,08	1,35 ± 0,29	N.S.
NADA	1,00 ± 0,42	0,31 ± 0,09	N.S.
OEA	1,00 ± 0,10	1,03 ± 0,10	N.S.
ETAEA	1,00 ± 0,55	1,56 ± 0,60	N.S.
20-hydroxy-PGF2a	1,00 ± 0,42	1,41 ± 0,52	N.S.
20-hydroxy-PGE2	1,00 ± 0,11	1,69 ± 0,24	N.S.
2,3-dinor-8-iso-PGF2a	1,00 ± 0,17	1,10 ± 0,21	N.S.
2,3-dinor-11B-iso-PGF2a	1,00 ± 0,11	1,39 ± 0,19	N.S.
20-carboxy-LTB4	1,00 ± 0,35	0,65 ± 0,17	N.S.
20-hydroxy-LTB4	1,00 ± 0,18	2,79 ± 1,19	**< 0.001**
iPF2a	1,00 ± 0,22	1,03 ± 0,27	N.S.
8-iso-15-R-PGF2a	1,00 ± 0,21	1,04 ± 0,13	N.S.

8-iso-PGF2a	1,00 ± 0,19	0,85 ± 0,13	N.S.
11beta-PGF2a	1,00 ± 0,06	1,03 ± 0,10	N.S.
PGF2a	1,00 ± 0,22	1,07 ± 0,10	N.S.
PGE3	1,00 ± 0,23	1,09 ± 0,09	N.S.
PGD3	1,00 ± 0,13	0,95 ± 0,14	N.S.
8-iso-PGE2	1,00 ± 0,11	1,58 ± 0,36	N.S.
PGE2	1,00 ± 0,10	1,47 ± 0,33	N.S.
11beta-PGE2	1,00 ± 0,10	1,32 ± 0,32	N.S.
PGD2	1,00 ± 0,14	1,57 ± 0,33	N.S.
TXB2	1,00 ± 0,62	0,67 ± 0,50	N.S.
5-iPF2a VI_1	1,00 ± 0,05	1,48 ± 0,25	N.S.
5-iPF2a VI_2	1,00 ± 0,05	1,48 ± 0,24	N.S.
8,12-iPF2a IV	1,00 ± 0,04	0,95 ± 0,10	N.S.
9,12,13-TriHOME	1,00 ± 0,27	1,02 ± 0,23	N.S.
9,10,13-TriHOME	1,00 ± 0,18	1,19 ± 0,31	N.S.
8-iso-13,14-dihydro-15-keto-PGF2a	1,00 ± 0,09	1,11 ± 0,11	N.S.
13,14dihydro-15-keto-PGF2a	1,00 ± 0,13	1,74 ± 0,36	N.S.
GUDCA	1,00 ± 0,03	1,15 ± 0,05	N.S.
13,14-dihydro-PGF2a	1,00 ± 0,18	1,32 ± 0,29	N.S.
GCA	1,00 ± 0,34	0,90 ± 0,17	N.S.
13,14-dihydro-15-keto-PGE2	1,00 ± 0,13	1,29 ± 0,26	N.S.
13,14-dihydro-15-keto-PGD2	1,00 ± 0,11	1,17 ± 0,16	N.S.
5S,6R-LipoxinA4	1,00 ± 0,11	1,46 ± 0,23	N.S.
5S,6S-LipoxinA4	1,00 ± 0,16	1,32 ± 0,20	N.S.
1a,1b-dihomo-PGF2a	1,00 ± 0,15	1,05 ± 0,14	N.S.
CA	1,00 ± 0,51	0,31 ± 0,11	N.S.
GCDCA	1,00 ± 0,01	1,05 ± 0,04	N.S.
GDCA	1,00 ± 0,03	1,06 ± 0,03	N.S.
bicyclo-PGE2	1,00 ± 0,17	1,18 ± 0,17	N.S.
12,13-DiHODE	1,00 ± 0,09	1,05 ± 0,13	N.S.
8S,15S-DiHETE	1,00 ± 0,25	1,31 ± 0,25	N.S.
10S,17S-DiHDoHE	1,00 ± 0,36	1,01 ± 0,35	N.S.

17,18-DiHETE	1,00 ± 0,03	1,00 ± 0,12	N.S.
14,15-DiHETE	1,00 ± 0,08	1,10 ± 0,22	N.S.
12,13-DiHOME	1,00 ± 0,08	0,67 ± 0,07	N.S.
9,10-DiHOME	1,00 ± 0,04	1,00 ± 0,15	N.S.
14,15-DiHETrE	1,00 ± 0,06	1,29 ± 0,34	N.S.
12S-HHTrE	1,00 ± 0,71	0,72 ± 0,51	N.S.
19,20-DiHDPA	1,00 ± 0,04	1,20 ± 0,22	N.S.
CDCA	1,00 ± 0,20	1,20 ± 0,18	N.S.
11,12-DiHETrE	1,00 ± 0,08	1,23 ± 0,31	N.S.
DCA	1,00 ± 0,37	0,46 ± 0,26	N.S.
9-HOTrE	1,00 ± 0,12	0,86 ± 0,08	N.S.
8,9-DiHETrE	1,00 ± 0,06	1,68 ± 0,38	N.S.
GLCA	1,00 ± 0,08	1,13 ± 0,06	N.S.
(+/-) 18-HEPE	1,00 ± 0,12	0,56 ± 0,05	N.S.
15S-HEPE	1,00 ± 0,07	1,28 ± 0,13	N.S.
20-HETE	1,00± 0,04	0,85 ± 0,08	N.S.
5,6-DiHETrE	1,00 ± 0,05	1,15 ± 0,06	N.S.
12(S)-HEPE	1,00 ± 0,36	1,37 ± 0,32	N.S.
(+/-) 9HEPE	1,00 ± 0,12	1,15 ± 0,23	N.S.
13-HODE	1,00 ± 0,09	1,03 ± 0,08	N.S.
12,13-EpOME	1,00 ± 0,10	0,68 ± 0,04	N.S.
5(S)-HEPE	1,00 ± 0,11	1,03 ± 0,13	N.S.
9-HODE	1,00 ± 0,10	1,14 ± 0,15	N.S.
9,10-EpOME	1,00 ± 0,09	1,02 ± 0,03	N.S.
(+/-) 17-HDoHE	1,00 ± 0,40	0,57 ± 0,08	N.S.
(+/-) 13-HDoHE	1,00 ± 0,05	1,22 ± 0,16	N.S.
(+/-) 7-HDoHE	1,00 ± 0,36	0,17 ± 0,01	N.S.
(+/-) 4-HDoHE	1,00± 0,08	1,09 ± 0,14	N.S.
19(20)-EpDPE	1,00 ± 0,09	1,14 ± 0,13	N.S.
(+/-) 20-HDoHE	1,00 ± 0,10	1,50 ± 0,23	N.S.
15-HETE	1,00 ± 0,06	1,41 ± 0,19	N.S.
14,15-EpETrE	1,00 ± 0,08	1,51 ± 0,29	N.S.

(+/-) 16-HDoHE	1,00 ± 0,13	1,36 ± 0,21	N.S.
16(17)-EpDPE	1,00 ± 0,10	1,71 ± 0,34	N.S.
11-HETE	1,00 ± 0,08	1,15 ± 0,16	N.S.
9-HETE	1,00 ± 0,14	1,14 ± 0,19	N.S.
11(12)-EpETrE	1,00 ± 0,03	1,30 ± 0,26	N.S.
(+/-) 14-HDoHE	1,00 ± 0,30	1,30 ± 0,25	N.S.
(+/-) 10-HDoHE	1,00 ± 0,08	1,42 ± 0,24	N.S.
12-HETE	1,00 ± 0,33	1,38 ± 0,41	N.S.
8-HETE	1,00 ± 0,16	0,98 ± 0,07	N.S.
8(9)-EpETrE	1,00 ± 0,22	0,79 ± 0,09	N.S.
(+/-) 11-HDoHE	1,00 ± 0,09	1,12 ± 0,11	N.S.
15(S)-HETrE	1,00 ± 0,10	1,10 ± 0,14	N.S.
(+/-) 8-HDoHE	1,00 ± 0,09	1,24 ± 0,13	N.S.
5-HETE	1,00 ± 0,08	0,92 ± 0,09	N.S.
8(S)-HETrE	1,00 ± 0,19	1,00 ± 0,14	N.S.
5(S)-HETrE	1,00 ± 0,08	0,64 ± 0,11	N.S.
Palmitic acid (16:0)	1,00 ± 0,20	0,67 ± 0,06	N.S.
Stearic aicd (18:0)	1,00 ± 0,25	0,69 ± 0,10	N.S.
Linoleic acid (18:2-ω6)	1,00 ± 0,20	0,50 ± 0,05	N.S.
α-Linolenic acid (18:3-ω3)	1,00 ± 0,21	0,41 ± 0,04	N.S.
γ-Linolenic acid (18:3-ω6)	1,00 ± 0,21	0,42 ± 0,04	N.S.
Dihomo-γ-linolenic acid (20:3-ω6)	1,00 ± 0,23	0,51 ± 0,06	N.S.
Mead acid (20:3-ω9)	1,00 ± 0,20	0,52 ± 0,07	N.S.
AA (20:4-ω6)	1,00 ± 0,34	0,43 ± 0,03	N.S.
Eicosapentaenoic acid (20:5-ω3)	1,00 ± 0,18	0,70 ± 0,10	N.S.
Docosapentaenoic acid (22:5-ω3)	1,00 ± 0,11	0,82 ± 0,09	N.S.
Docosatetraenoic acid (22:4-ω6)	1,00 ± 0,21	0,74 ± 0,07	N.S.
Docosahexaenoic acid (22:6-ω3)	1,00 ± 0,11	0,66 ± 0,05	N.S.
S-1-P 16(1)	1,00 ± 0,07	0,80 ± 0,09	N.S.
S-1-P 18(0)	1,00 ± 0,06	0,99 ± 0,05	N.S.
S-1-P 18(1)	1,00 ± 0,03	1,02 ± 0,03	N.S.
S-1-P 18(2)	1,00 ± 0,03	0,88 ± 0,04	N.S.

LPE (14:0)	1,00 ± 0,12	0,94 ± 0,12	N.S.
LPE (16:0)	1,00 ± 0,03	1,24 ± 0,09	N.S.
LPE (16:1)	1,00 ± 0,10	0,90 ± 0,10	N.S.
LPE (18:0)	1,00 ± 0,10	1,18 ± 0,21	N.S.
LPE (18:1)	1,00 ± 0,02	1,07 ± 0,04	N.S.
LPE (18:2)	1,00 ± 0,05	1,01 ± 0,04	N.S.
LPE (18:3)	1,00 ± 0,11	1,05 ± 0,07	N.S.
LPE (20:3)	1,00 ± 0,04	0,99 ± 0,10	N.S.
LPE (20:4)	1,00 ± 0,04	1,23 ± 0,08	N.S.
LPE (20:5)	1,00 ± 0,06	1,28 ± 0,10	N.S.
LPE (22:4)	1,00 ± 0,04	0,95 ± 0,09	N.S.
LPE (22:5)	1,00 ± 0,04	0,86 ± 0,12	N.S.
LPE (22:6)	1,00 ± 0,06	1,23 ± 0,10	N.S.
LPI 14(0)	1,00 ± 0,08	0,67 ± 0,04	N.S.
LPI 16(0)	1,00 ± 0,10	0,89 ± 0,07	N.S.
LPI 16(1)	1,00 ± 0,05	0,73 ± 0,02	N.S.
LPI 18(0)	1,00 ± 0,07	1,13 ± 0,11	N.S.
LPI 18(1)	1,00 ± 0,11	0,74 ± 0,05	N.S.
LPI 18(2)	1,00 ± 0,08	0,80 ± 0,06	N.S.
LPI 20(4)	1,00 ± 0,04	1,21 ± 0,12	N.S.
LPI 22(4)	1,00 ± 0,10	0,91 ± 0,03	N.S.
LPI 22(6)	1,00 ± 0,20	0,82 ± 0,02	N.S.
LPA 14(0)	1,00 ± 0,13	0,77 ± 0,11	N.S.
LPA 16(0)	1,00 ± 0,04	0,92 ± 0,09	N.S.
LPA 16(1)	1,00 ± 0,09	0,73 ± 0,10	N.S.
LPA 18(0)	1,00 ± 0,06	0,90 ± 0,10	N.S.
LPA 18(1)	1,00 ± 0,05	0,76 ± 0,03	N.S.
LPA 18(2)	1,00 ± 0,08	0,84 ± 0,06	N.S.
LPA 20(3)	1,00 ± 0,02	0,80 ± 0,07	N.S.
LPA 20(4)	1,00 ± 0,06	0,93 ± 0,05	N.S.
LPA 20(5)	1,00 ± 0,09	0,94 ± 0,05	N.S.
LPA 22(4)	1,00 ± 0,07	0,77 ± 0,21	N.S.

LPA 22(6)	1,00 ± 0,06	1,07 ± 0,08	N.S.
LPG 22(6)	1,00 ± 0,03	1,37 ± 0,07	N.S.
LPG 22(4)	1,00 ± 0,08	1,02 ± 0,05	N.S.
LPG 20(5)	1,00 ± 0,23	0,52 ± 0,14	N.S.
LPG 20(4)	1,00 ± 0,02	0,94 ± 0,04	N.S.
LPG 20(3)	1,00 ± 0,14	1,02 ± 0,10	N.S.
LPG 18(3)	1,00 ± 0,12	1,05 ± 0,06	N.S.
LPG 18(2)	1,00 ± 0,07	1,15 ± 0,02	N.S.
LPG 18(1)	1,00 ± 0,08	0,85 ± 0,06	N.S.
LPG 18(0)	1,00 ± 0,06	1,14 ± 0,15	N.S.
LPG 16(1)	1,00 ± 0,06	0,67 ± 0,04	N.S.
LPG 16(0)	1,00 ± 0,08	0,67 ± 0,05	N.S.
LPG 14(0)	1,00 ± 0,15	0,57 ± 0,04	N.S.
LPS 16(0)	1,00 ± 0,08	1,07 ± 0,18	N.S.
LPS 18(0)	1,00 ± 0,19	1,24 ± 0,30	N.S.
LPS 18(1)	1,00 ± 0,48	1,53 ± 0,06	N.S.
LPS 18(2)	1,00 ± 0,31	0,90 ± 0,10	N.S.
LPS 20(4)	1,00 ± 0,24	1,01 ± 0,11	N.S.
LPS 22(4)	1,00 ± 0,11	1,00 ± 0,13	N.S.
LPS 22(6)	1,00 ± 0,19	1,12 ± 0,14	N.S.
cLPA 18(0)	1,00 ± 1,00	0,00 ± 0,00	N.S.
cLPA 18(1)	1,00 ± 1,00	0,00 ± 0,00	N.S.
cLPA 18(2)	1,00 ± 1,00	0,00 ± 0,00	N.S.
cLPA 20(4)	1,00 ± 1,00	0,00 ± 0,00	N.S.
TCA	1,00 ± 0,56	2,80 ± 1,03	**< 0.001**
GCDCA	1,00 ± 0,43	1,30 ± 0,33	N.S.
GDCA	1,00 ± 0,13	0,75 ± 0,20	N.S.
GCA	1,00 ± 0,72	0,87 ± 0,37	N.S.
THDCA	1,00 ± 0,49	3,51 ± 1,37	**< 0.001**
TDCA	1,00 ± 0,54	3,29 ± 1,46	**< 0.001**
TCDCA	1,00 ± 0,52	0,81 ± 0,20	N.S.
CUDA	1,00 ± 0,03	1,02 ± 0,03	N.S.

TLCA	1,00 ± 0,48	0,76 ± 0,19	N.S.
LCA-3S	1,00 ± 0,19	1,06 ± 0,31	N.S.

Values are expressed relative to the Control and represent the means±SEM of 4/5 plasma pools. N.S., not significant
